# Retinal autofluorescence findings after COVID-19

**DOI:** 10.1186/s40942-021-00341-5

**Published:** 2021-11-27

**Authors:** Paula M. Marinho, Alléxya A. A. Marcos, Ana M. C. Branco, Walid M. Mourad, Victoria Sakamoto, Andre C. Romano, Michel Farah, Richard B. Rosen, Paulo Schor, Paulo Abraao, Heloisa Nascimento, Rubens Belfort

**Affiliations:** 1 São Paulo Hospital, Paulista School of Medicine, Federal University of São Paulo, Rua Botucatu 816 Vila Clementino, São Paulo, Brazil; 2 Vision Institute – IPEPO, São Paulo, Brazil; 3 Young Leadership Program, National Academy of Medicine, Rio de Janeiro, Brazil; 4 Santo Amaro University, São Paulo, Brazil; 5 New York Eye and Ear Infirmary of Mount Sinai, New York, NY USA

**Keywords:** Coronavirus, SARS-CoV-2 disease, Eye, Optical coherence tomography, Autofluorescence

## Abstract

The main purpose of this study was to investigate the presence of retinal autofluorescence findings in COVID-19 patients. Observational study conducted in São Paulo in 2020. Demographic, medical history, and concomitant events, as well as medications used, hospitalization details, and laboratory test results, were obtained. Patients underwent eye examination and multimodal imaging, including color, red-free, autofluorescence fundus photography and optical coherence tomography. Eighteen patients had autofluorescence findings (6 females; average age 54 years, range 31 to 86 years; 26 eyes). Hyper-autofluorescence findings were present in 6 patients, Hypo-autofluorescence in 14 patients, and 6 patients had mixed pattern lesions. Retinal autofluorescence abnormalities were present in COVID-19 patients and may be secondary to primary or secondary changes caused by the SARS-CoV-2.

## Introduction

Early clinical evidence suggests that cases of COVID-19 are frequently characterized by increased inflammation, renin-angiotensin-aldosterone system (RAS) imbalance, and a particular form of vasculopathy, thrombotic microangiopathy, and intravascular coagulopathy [1].

The retina could be affected either by direct tissue damage from SARS-CoV-2 and its immunogenicity or by thrombotic complications [2, 3]. Primary or secondary retinal abnormalities mostly related to vascular structures have been reported on multimodal imaging studies [4–6].

Fundus autofluorescence (FAF) imaging provides a topographic mapping of lipofuscin distribution in the retinal pigment epithelium (RPE) cell monolayer, and other fluorophores occur with the outer retina and the sub-neurosensory space [7]. This study aims to investigate FAF findings in COVID-19 patients.

## Methods

The study was approved by the institutional and national ethics research committees (Research Ethics Committee of Federal University of Sao Paulo UNIFESP #30725020.8.0000.5505 and INVITARE Pesquisa Clínica Auditoria e Consultoria Institutional Review Board Ethics Committee number 3.975.953). All patients or their representatives agreed to participate.

We conducted a observational study evaluating outpatients with confirmed COVID-19 diagnosis based on positive antibody tests (immunoglobulin G and immunoglobulin M titers) or PCR (using nasal/oral swabs). Patients with previous ophthalmological history and patients for whom fundus exam was impossible were excluded.

Demographic and clinical information covering medical history, concomitant medical events and medications, hospitalization details, and laboratory tests were obtained. Ophthalmic examination included measurement of best-corrected visual acuity (BCVA), Goldman applanation tonometry (IOP), and both anterior and posterior biomicroscopy. Binocular indirect fundus examination and color, red-free, and autofluorescence fundus photography were performed (Topcon DRI-OCT Triton Swept-source OCT, and California Optos®). Optical coherence tomography (OCT) imaging included: Angio Retina 3.0 mm^2^; HD Angio Retina 6.0 mm^2^; Enhanced HD Line; Cross Line; Raster; Radial Lines; Ganglion cell complex (GCC) (Optovue RTVue-XR Avanti®).

The data were analyzed using the STATA 14.0 program (StataCorp LP, College Station, TX, USA). Frequency tables were used for descriptive analyses.

## Results

In late 2020, as part of the eye examination of a group of 106 patients, 18 patients with FAF changes were identified. The average time between diagnosis and the first eye exam was 44 days (±22 days), we’ve considered this time frame to start with symptoms onset. None of the patients had a previous ophthalmologic history, specially concerning previous ocular inflammation. All patients were evaluated at a convalescence period and disease severity ranged from mild to severe. We have considered severe cases patients whom required mechanical ventilatory support, moderate cases the ones whom required hospitalization but non-invasive ventilation and mild cases the ones without hospitalization. Of the 18 patients, 12 required previous hospital admission and were examined after hospital discharge. Table 1 presents data regarding epidemiology and clinical examination.

**Table 1:** Patients demography (n = 18)

	Median ± SD
Age	54 ± 15 years
Female	6 (33%)
BCVA OD	0.15 ± 0.25 (20/28)
BCVA OS	0.09 ± 0.17 (20/24)
Days between symptoms onset and evaluation	44. (±24 days)
Type 2 diabetes	2 (11%)
High blood pressure	6 (33%)
Diabetes and high blood pressure concomitantly	2 (11%)

**Fig. 1:**
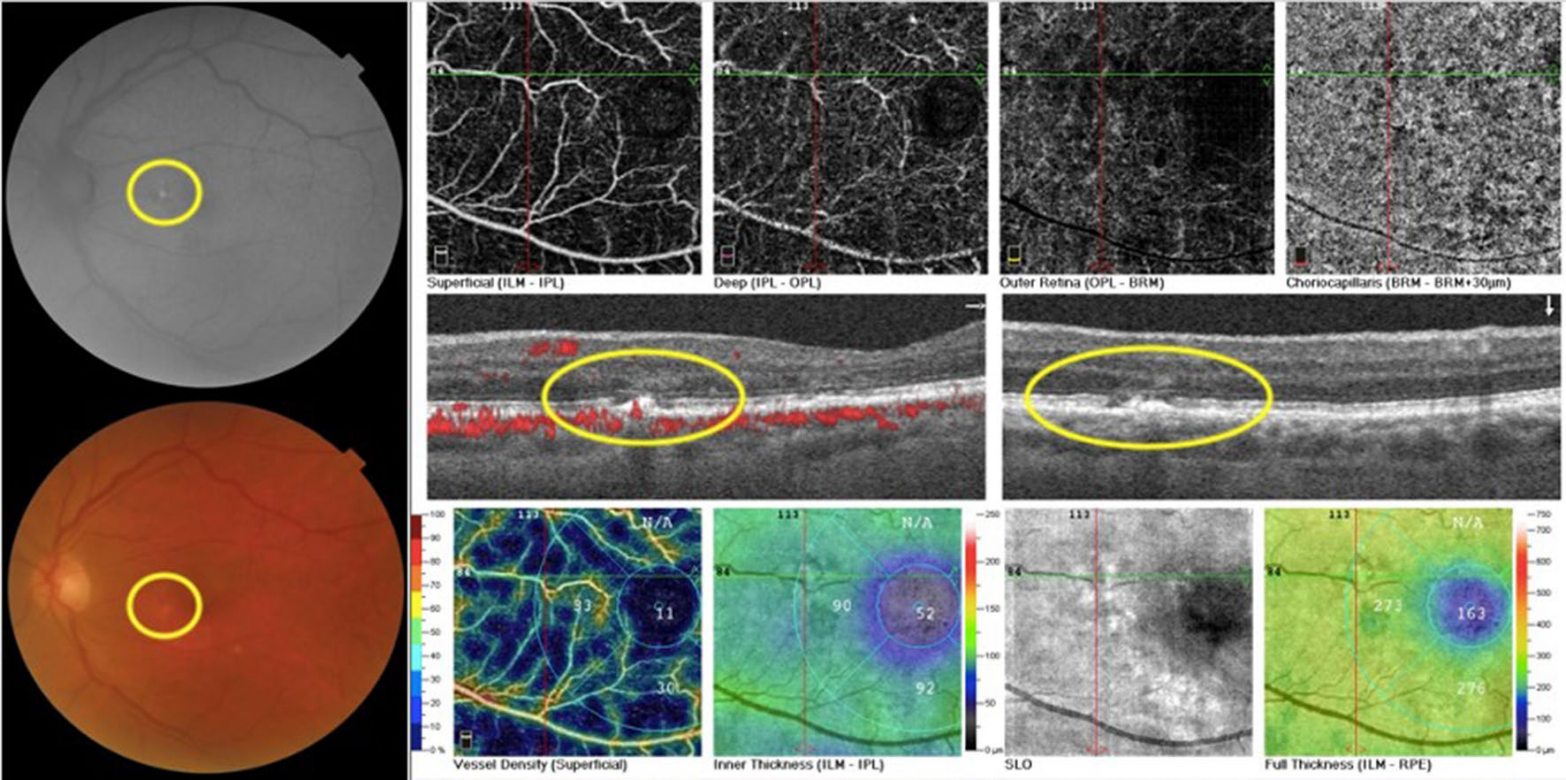
Composite of the left eye of patient 7.  Composite depicts a hypopigmented area in the papillomacular bundle more evident as a hyper-autofluorescent lesion on FAF. OCT B-scan of the lesion displays RPE irregularity with adjacent cell loss in the ellipsoid zone

**Fig. 2:**
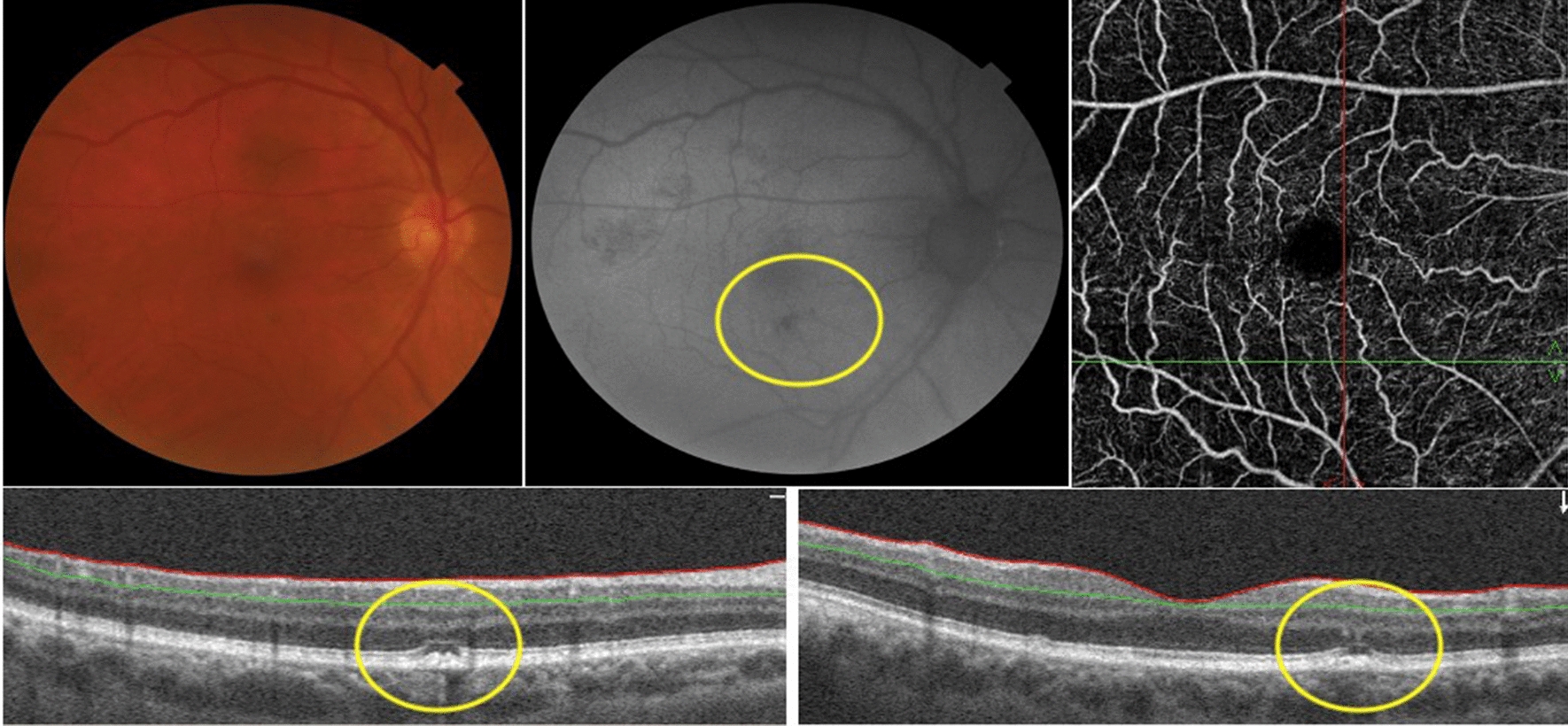
Composite of the right eye of patient 7.  Image shows a hypopigmented area in the papillomacular bundle more evident as a hypo-autofluorescent lesion on FAF. OCT B-scan of the lesion displays RPE irregularity with adjacent cell loss in the ellipsoid zone

Among the 18 patients, 10 had findings only in one eye and 8 in both eyes. Most of these were depicted at posterior pole (16 eyes) and 4 other eyes had alterations contiguous to the optic nerve. Hyper-autofluorescence (HyperFAF) (Fig. 1) was present in 6 eyes of 5 patients (27.8%–5/18), and one eye presented with uniquely HyperFAF. OCT of those areas was associated with the outer retina findings, mainly in the interdigitation and ellipsoid zones (Fig. 1). Hypo-autofluorescence (HypoFAF) (Fig. 2) was present in 18 eyes of 14 patients (77.8%–14/18). OCT of those areas was associated with outer retina cell loss and RPE elevation. One eye also presented with subretinal fluid.

**Fig. 3:**
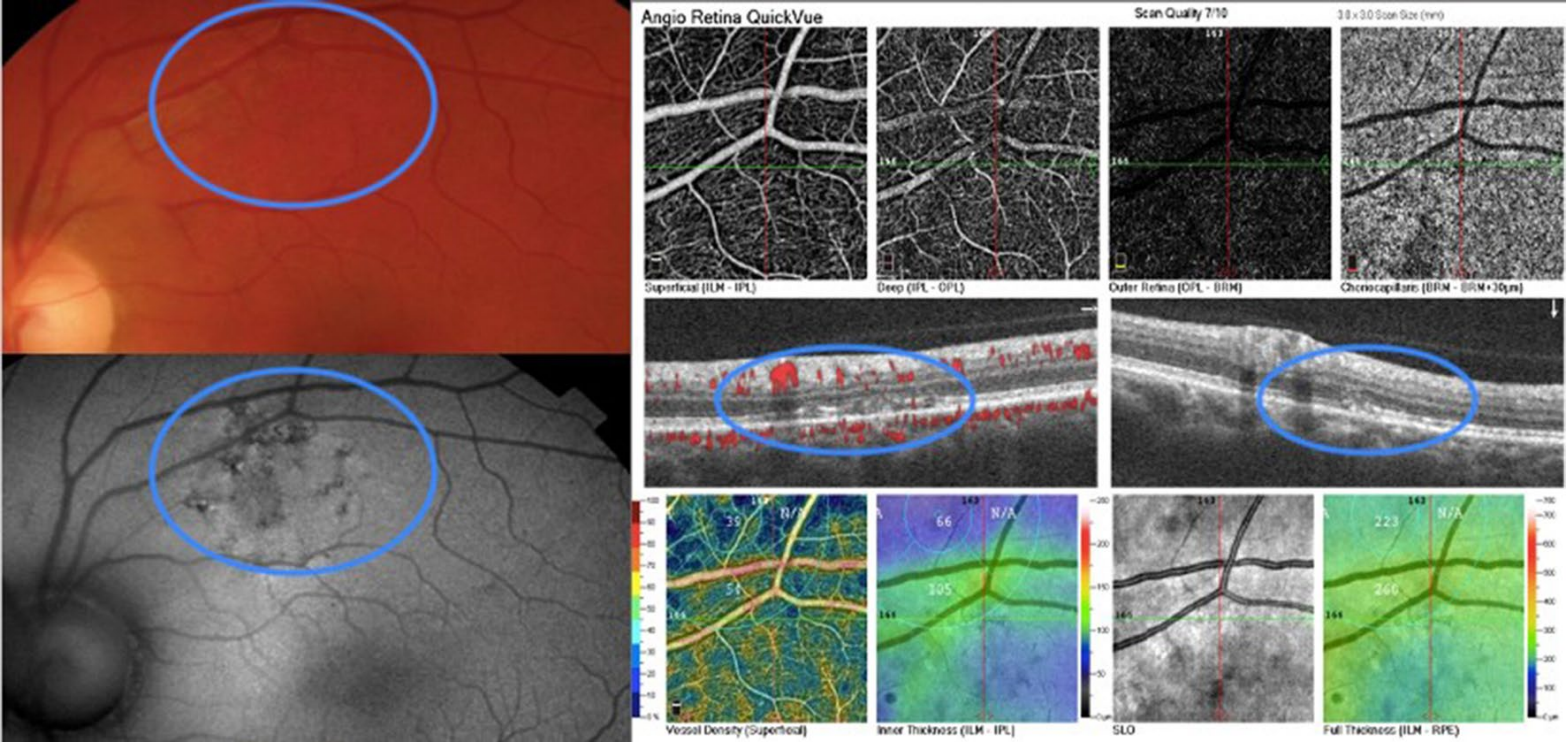
Composite of the left eye of patient 6. FAF shows a retinal alteration more prominent at FAF with a mixed pattern of hyper-hypoFAF. OCT B-scan of the area displays disruption of the interdigitation zone, outer segment layer, and ellipsoid zone

**Fig. 4:**
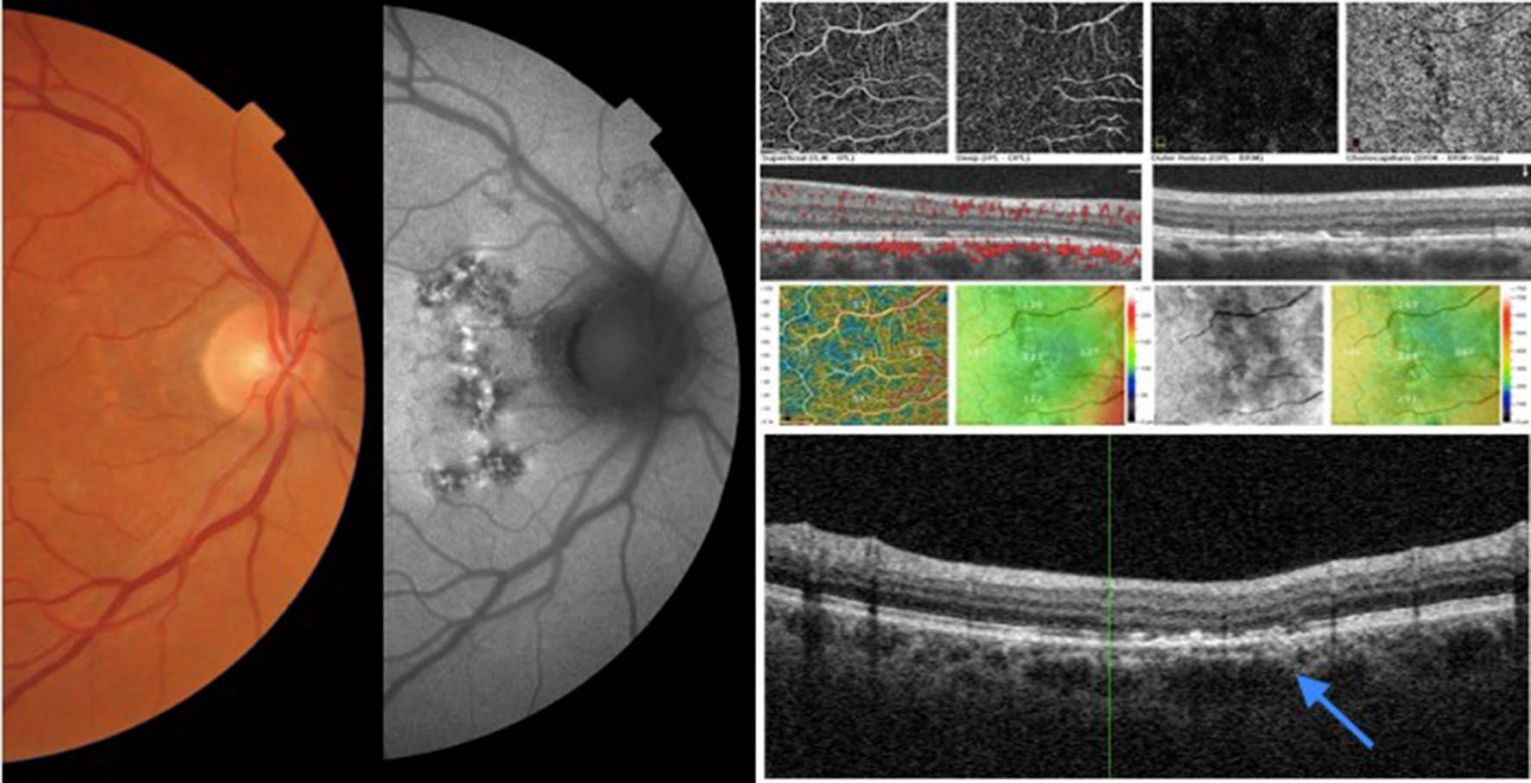
Composite of the right eye of patient 10. This composite shows a retinal alteration more prominent at FAF with a mixed pattern of hyper-hypoFAF. OCT B-scan displays retinal thinning due to loss of external retina layers and outer nuclear layer

Seven eyes of 6 patients (33.3%–6/18) showed mixed patterns of hyper-autofluorescence and hypo-autofluorescence. These findings were predominantly seen adjacent to vascular structures, especially veins, in different retinal areas (Figs. 3 and 4). OCT findings of retinal thinning were associated with a disturbance of the ellipsoid zone, photoreceptors outer segments, and interdigitation zone (Figs. 3 and 4).

Table 2 presents the correlation between autofluorescence patterns, OCT findings and patient data.

**Table 2:** Correlation between autofluorescence patterns, OCT findings and patient data

	Age (Years)	Comorbidities	FAF patterns		OCT Findings		Hospitalization	IOT	Anticoagulation	Antibiotic	D-dimer
			Right eye	Left eye	Right eye	Left eye					
1	51	None	HypoFAF	HypoFAF	RPE irregularity	RPE elevation	N	N	N	N	N
2	82	None	Hypo-hyper-FAF	Isolated areas of HypoFAF and HyperFAF	Choroidal irregularity with area of RPE disruption and subretinal fluid	Area of cellular loss at the level of the ellipsoid zone	Y	N	–	–	–
3	51	None	HypoFAF	None	Cystoid spaces in the interdigitation zone	None	N	N	N	N	N
4	63	High blood pressure and diabetes	None	HypoFAF	None	Cellular loss at ellipsoid zone	Y	Y	Y	N	1.20 ng/mL
5	53	None	Isolated areas of HyperFAF and isolated areas of HypoFAF	Isolated areas of HyperFAF and isolated areas of HypoFAF	Disruption of the interdigitation zone	Disruption of the interdigitation zone with local retinal thinning	Y	N	N	Y	0.45 ng/mL
6	86	None	Hypo-hyper-FAF	Hypo-hyper-FAF	Disruption of the interdigitation zone, outer segment layer and ellipsoid zone	Disruption of the interdigitation zone, outer segment layer and ellipsoid zone	Y	N	N	Y	1.63 ng/mL
7	51	High blood pressure and diabetes	HypoFAF	HyperFAF	RPE irregularity with subretinal liquid and disruption of the ellipsoid zone	RPE irregularity with adjacent cell loss at the ellipsoid zone	Y	N	N	N	0.53ng/mL
8	69	None	Hypo-hyper-FAF	HypoFAF	RPE elevation	RPE irregularity	Y	N	N	Y	N
9	66	High blood pressure	HypoFAF	None	External limiting membrane irregularity	None	Y	N	Y	Y	0.61 ng/mL
10	49	None	Hypo-hyper-FAF	HypoFAF	Retinal thinning and loss of external retina and outer nuclear layers	RPE elevation and outer retinal layer loss	No	No	No	No	–
11	57	High blood pressure	None	Hypo-hyper-FAF	None	Retinal thinning due to loss of external retinal and outer nuclear layers	Y	N	N	Y	0.71 ng/mL
12	62	High blood pressure	HypoFAF	None	Retinal Pigmented Epithelial Detachment and adjacent loss of ellipsoid zone	None	Y	N	Y	N	–
13	48	None	Isolated areas of HyperFAF and HypoFAF	None	RPE elevation	None	Y	N	–	–	0.85 ng/mL
14	31	None	None	HypoFAF	None	Disruption of the interdigitation and outer segment layers and adjacent scarring	--	--	N	N	N
15	35	None	HypoFAF	None	None	None	N	N	N	Y	N
16	58	High blood pressure	HypoFAF, with surrounding area of HyperFAF	None	Disruption of external retina with adjacent scarring	None	Y	N	Y	Y	0.93 ng/mL
17	34	None	HypoFAF	HypoFAF	RPE and ellipsoid irregularity	RPE irregularity and elevation	Y	N	N	N	1.80 ng/mL
18	41	None	Hypo-hyper-FAF	None	Choroidal elevetaion	None	N	N	N	N	N

## Discussion

FAF is a non-invasive diagnostic tool that documents the metabolic status of lipofuscin levels throughout the eye’s posterior pole. It can be a helpful marker of outer retinal health helpful in monitoring various ophthalmic conditions, including uveitis or photoreceptor diseases. HyperFAF patterns are often related to active retinal pigmentary epithelium inflammation, and HypoFAF patterns are found in chronic and scar lesions.

The FAF patterns reported here appeared similar to those previously described in other diseases, such as syphilis [8], tuberculosis [9], inflammatory maculopathies [10], and even age-related macular degeneration [11] and may implicate pathogenic mechanisms. Our small sample prevented us the use of multiple logistic regressions to assess whether comorbidities, treatment performed, or changes in laboratory tests were related to the ophthalmological findings. Since the descriptive nature of the study aiming to report the FAF findings in patients its valuable to emphasize the multiple confusing factors, specially the heterogeneity of the population involved.

Fundus autofluorescence results from the interaction between natural fluorophores and the adjacent tissues, and variety of clinical COVID-19 presentations [12–15] can explain the broad spectrum of findings [12]. Schmitz-Valckenberg et al. have previously reported that inflammatory diseases may present different pattern of FAF over time [16], and according to the affected area, it can appear hypo-autofluorescent early and mixed later on.

Previous publications have reported retinal findings in COVID19 patients [5, 12–15, 17, 18]. FAF alterations have been presented among case reports [17–19] and the frequency has increased since the beginning of the pandemic. To our knowledge this is the biggest number of cases congregated and, in face of a new and still poorly understood disease, a more detailed analysis of the RPE-choriocapillaris complex may contribute to the better understand of COVID-19 pathophysiology in the eye ant it’s presumed effect, bring new light in it’s pathophysiology. The high prevalence of a hyper-hypo-autofluorescence pattern near vascular structures suggests that vessels may be preferentially affected, which agrees with other studies suggesting a vascular component to the SARS-CoV-2 pathogenesis [20, 21].

## Conclusions

Autofluorescence may be an useful resource to detect lesions otherwise missed. The presence of hyper-autofluorescence speaks in favor of acuter lesions and towards a somewhat neglected RPE-choriocapillaris complex disfunction. Further investigation is mandatory to better understand the pathophysiology and presumed long term implications.

## Data Availability

The datasets used and/or analysed during the current study are available from the corresponding author on reasonable request.

## References

[1] Henry BM, Vikse J, Benoit S, Favaloro EJ, Lippi G (2020). Hyperinflammation and derangement of renin-angiotensin-aldosterone system in COVID-19: a novel hypothesis for clinically suspected hypercoagulopathy and microvascular immunothrombosis. Clinica Chimica Acta.

[2] Klok FA, Kruip MJHA, van der Meer NJM, Arbous MS, Gommers DAMPJ, Kant KM (2020). Incidence of thrombotic complications in critically ill ICU patients with COVID-19. Thromb Res.

[3] Wu Y, Xu X, Chen Z, Duan J, Hashimoto K, Yang L (2020). Nervous system involvement after infection with COVID-19 and other coronaviruses. Brain Behav Immun.

[4] Costa ÍF, Bonifácio LP, Bellissimo-Rodrigues F, Rocha EM, Jorge R, Bollela VR (2021). Ocular findings among patients surviving COVID-19. Sci Rep.

[5] Marinho PM, Marcos AAA, Romano AC, Nascimento H, Belfort R (2020). Retinal findings in patients with COVID-19. Lancet.

[6] Marinho PM, Nascimento H, Marcos AAA, Romano AC, Rosen RB, Belfort R. Reply to Editorial: Interpretation of OCT and fundus findings in COVID-19 patients in recent Lancet publication. Eye. 2020; http://www.nature.com/articles/s41433-020-01283-2. Accessed 15 Jul 2021. 10.1038/s41433-020-01283-2 PMC7687980 33239764

[7] Schmitz-Valckenberg S, Holz FG, Bird AC, Spaide RF (2008). Fundus autofluorescence imaging: review and perspectives. Retina.

[8] Matsumoto Y, Spaide RF (2007). Autofluorescence imaging of acute syphilitic posterior placoid chorioretinitis. Retin Cases Brief Rep.

[9] Samy A, Lightman S, Ismetova F, Talat L, Tomkins-Netzer O (2014). Role of autofluorescence in inflammatory/infective diseases of the retina and choroid. J Ophthalmol.

[10] Yeh S, Lee A, Forooghian F, Bergstrom C, Yan J, Lee C (2014). Fundus autofluorescence features in the inflammatory maculopathies. Clin Ophthalmol.

[11] Yung M, Klufas MA, Sarraf D (2016). Clinical applications of fundus autofluorescence in retinal disease. Int J Retin Vitr.

[12] Pereira LA, Soares LCM, Nascimento PA, Cirillo LRN, Sakuma HT, da Veiga GL (2020). Retinal findings in hospitalised patients with severe COVID-19. Br J Ophthalmol..

[13] Gascon P, Briantais A, Bertrand E, Ramtohul P, Comet A, Beylerian M (2020). Covid-19-associated retinopathy: a case report. Ocul Immunol Inflamm..

[14] Zago Filho LA, Lima LH, Melo GB, Zett C, Farah ME (2020). Vitritis and outer retinal abnormalities in a patient with COVID-19. Ocular Immunol Inflamm.

[15] Gaba WH, Ahmed D, Al Nuaimi RK, Dhanhani AA, Eatamadi H. Bilateral central retinal vein occlusion in a 40-year-old man with severe coronavirus disease 2019 (COVID-19) pneumonia. Am J Case Rep. 2020;21. https://www.amjcaserep.com/abstract/index/idArt/927691. Accessed 15 July 2021. 10.12659/AJCR.927691 PMC7603800 33116072

[16] R. S. Chorioretinal Inflammatoy Disorders. In: Holz FG, Schmitz-Valckenberg S, Spaide RF, Bird AC, editors. Atlas of fundus autofluorescence imaging: with 1 table. Berlin Heidelberg: Springer; 2007:207–239.

[17] Duff SM, Wilde M, Khurshid G. Branch retinal vein occlusion in a COVID-19 positive patient. Cureus. 2021; https://www.cureus.com/articles/51955-branch-retinal-vein-occlusion-in-a-covid-19-positive-patient. Accessed 15 July 2021. 10.7759/cureus.13586 PMC8009444 33815989

[18] Olguín-Manríquez F, Cernichiaro-Espinosa L, Olguín-Manríquez A, Manríquez-Arias R, Flores-Villalobos EO, Kawakami-Campos PA (2021). Unilateral acute posterior multifocal placoid pigment epitheliopathy in a convalescent COVID-19 patient. Int J Retin Vitr.

[19] de Souza EC, de Campos VE, Duker JS (2021). Atypical unilateral multifocal choroiditis in a COVID-19 positive patient. Am J Ophthalmol Case Rep.

[20] Siddiqi HK, Libby P, Ridker PM (2021). COVID-19—a vascular disease. Trends in Cardiovasc Med.

[21] Connors JM, Levy JH (2020). COVID-19 and its implications for thrombosis and anticoagulation. Blood.

